# Prognostic value of quickSOFA as a predictor of 28-day mortality among febrile adult patients presenting to emergency departments in Dar es Salaam, Tanzania

**DOI:** 10.1371/journal.pone.0197982

**Published:** 2018-06-14

**Authors:** Noémie Boillat-Blanco, Zainab Mbarack, Josephine Samaka, Tarsis Mlaganile, Aline Mamin, Blaise Genton, Laurent Kaiser, Thierry Calandra, Valérie D’Acremont

**Affiliations:** 1 Swiss Tropical and Public Health Institute, Basel, Switzerland; 2 University of Basel, Basel, Switzerland; 3 Ifakara Health Institute, Dar es Salaam, United Republic of Tanzania; 4 Infectious Diseases Service, University Hospital of Lausanne, Lausanne, Switzerland; 5 Mwananyamala Hospital, Dar es Salaam, United Republic of Tanzania; 6 Laboratory of Virology, Infectious Diseases Service, University of Geneva Hospitals, Geneva, Switzerland; 7 Department of Ambulatory Care and Community Medicine, University Hospital of Lausanne, Lausanne, Switzerland; 8 University of Geneva Medical School, Geneva, Switzerland; VU university medical center, NETHERLANDS

## Abstract

**Background:**

Quick Sequential Organ Failure Assessment (qSOFA) is a three-item clinical instrument for bedside identification of sepsis patients at risk of poor outcome. qSOFA could be a valuable triage tool in emergency departments of low-income countries, yet its performance in resource-limited settings remains unknown. The prognostic accuracy of qSOFA for 28-day all-cause mortality in febrile adults treated at the EDs in a low-income country was evaluated.

**Methods:**

Retrospective analysis of a prospective cohort study of consecutive patients (≥18 years) with fever (tympanic temperature ≥38°C and fever ≤7 days) who presented between July 2013 and May 2014 at four emergency departments in Dar es Salaam, Tanzania. Medical history, clinical examination, laboratory and microbiological data were collected to document the cause of fever. Variables for the previous and new sepsis criteria were collected at inclusion and qSOFA, SOFA and SIRS were measured at inclusion. Patients were followed up by phone at day 28. The performance (sensitivity, specificity and area under the receiver operating curve [AUROC]) of qSOFA (score ≥2), SOFA (increase of ≥2 points) and SIRS (≥2 criteria) as predictors of 28-day all-cause mortality was evaluated.

**Results:**

Among the 519 patients (median age: 30 years) included in the analysis, 47% were female and 25% were HIV positive. Overall, 85% had a microbiologically and/or clinically documented infection and 15% a fever of unknown origin. The most common site and causes of infections were the respiratory tract (43%), dengue (26%), malaria (6%) and typhoid fever (5%). Twenty-eight-day all-cause mortality was 6%: 3% for patients with a qSOFA <2 and 24% for those with a score ≥2 (absolute difference, 21%; 95% CI 12%-31%). The prognostic accuracy of qSOFA (AUROC 0.80, 95% CI 0.73–0.87) for 28-day mortality was similar to SOFA (AUROC 0.79, 0.71–0.87; p = 0.1) and better than SIRS (AUROC 0.61, 0.52–0.71; p<0.001).

**Conclusions:**

Among patients with fever at emergency departments in Tanzania, qSOFA had a prognostic accuracy for 28-day mortality comparable to SOFA and superior to SIRS. These results support the use of qSOFA as a triage tool to identify patients with sepsis and at risk of poor outcome in resource-limited countries.

**Trial registration:**

Clinicaltrials.gov Identifier: NCT01947075

## Introduction

Fever is a frequent medical condition leading to health care seeking and hospital admission [1–3]. Identification of patients with fever who have or are at risk of sepsis is a challenge in emergency departments, especially in low-income countries where resources are limited in term of healthcare workers and laboratory facilities. Early recognition of sepsis patients is critical as prompt initiation of appropriate therapy improves survival [4, 5].

Together with the publication of the Third International Consensus Definitions for Sepsis and Septic Shock (Sepsis-3) [6], a simplified clinical Sequential (Sepsis-related) Organ Failure Assessment (SOFA) score coined quick SOFA (qSOFA) derived from and validated in large clinical data sets, was proposed to help clinicians identify sepsis patients among those with suspected infection [4]. Composed of three easy-to-measure clinical parameters (i.e. Glasgow coma score, systolic blood pressure and respiratory rate), qSOFA exhibited higher predictive validity for in-hospital mortality than SOFA and Systemic Inflammatory Response Syndrome (SIRS) among encounters with suspected infection outside the ICU. In a recent prospective study conducted in emergency departments of four European countries, qSOFA had greater prognostic accuracy for in-hospital mortality than SIRS or severe sepsis, but not SOFA [7]. In other mostly retrospective studies performed in emergency departments, qSOFA was associated with in-hospital mortality, had an adequate to good predictive accuracy for mortality and had a low sensitivity but a high specificity to predict mortality [8–12].

On a large American observational study conducted in emergency departments and wards, commonly used early warning scores such as the Modified Early Warning Score (MEWS) and the National Early Warning Score (NEWS) were shown to be more accurate than the qSOFA score for predicting mortality [13]. These scores are more complicated and the NEWS needs oxygen saturation as a parameter. They have been designed in high-income countries and may be difficult to apply in a resource-limited environment.

Altogether, there is ample evidence derived from a growing number of studies suggesting that qSOFA may be a useful triage tool for prompt identification of sepsis patients in emergency departments. Given that it is composed of three clinical parameters rapidly and easily assessable at the bedside, it is a highly attractive instrument to be used in emergency departments of low-income and resource-limited countries, yet its performance in these settings remains unknown [14].

The objective of the present study was to evaluate the prognostic accuracy of qSOFA for 28-day all-cause mortality in febrile adult patients treated at emergency departments in Dar es Salaam, Tanzania and to compare it with SOFA and SIRS.

## Material and methods

### Study design, setting and population

A prospective cohort study to document the etiologies of fever was performed between July 18 2013 and May 14 2014 at the emergency departments of Mwananyamala Regional Hospital and connected health care facilities (Sinza Hospital, Magomeni Health Centre, Tandale Health Centre) in Kinondoni, the most populated (1,800,000 inhabitants) district of Dar es Salaam, the largest city (4,365,000 inhabitants) and economic center of Tanzania. This study gave a unique opportunity to evaluate qSOFA in this population. All consecutive adult (age ≥18 years) patients presenting with fever (tympanic temperature ≥38.0°C) at the emergency departments were prospectively screened for inclusion in the study. The inclusion criteria were: 1) the presence of a new fever defined as a tympanic temperature ≥38.0°C lasting for 7 days or less, 2) first consultation for the present problem. Exclusion criteria were: 1) refusal of HIV testing, 2) injury or trauma as main reason for consultation, 3) hospital admission within the last month (in order to include community-acquired infections), 4) delivery within 6 weeks. Clinical outcome was assessed by a visit or a phone call on day 7 and by a phone call on day 28.

### Study procedures

#### Data collection

Demographic characteristics, co-morbidities, symptoms and signs were collected at inclusion. Blood pressure was measured with an automated device (Omron^®^ M6).

#### Laboratory investigations

Complete blood count (Horiba Medical ABX Pentra 80 hematology analyzer), serum alanine transferase and serum creatinine (Biochemical Systems International fully automated clinical chemistry analyzer) were measured in all patients. Bilirubin (Biochemical Systems International fully automated clinical chemistry analyzer) was measured in a subgroup of patients who were admitted. Blood cultures were drawn on site in all patients and sent to the research laboratory of Ifakara Health Institute in Bagamoyo, Tanzania. Rapid diagnostic tests for HIV (Alere Determine^™^ HIV-1/2, confirmed by Trinity Biotech Uni-gold^™^ Recombigen^®^ HIV-1/2), dengue (SD BIOLINE Dengue Duo^®^), malaria (ICT Malaria P.f.^®^), typhoid fever (Reszon Diagnostics International TYPHIDOT Rapid IgM^®^) were performed on site on the day of inclusion in all patients. Additional diagnostic tests were performed using predefined algorithms adapted from a previously published study (S1 Table) [15].

#### Definitive diagnosis

Sites and causes of infections were established for each patient on the basis of predefined clinical and microbiological criteria derived from World Health Organization, Infectious Diseases Society of America guidelines and European Society of Clinical Microbiology and Infectious Diseases guidelines as defined previously [15]. The diagnostic workup in the emergency department was done by the study team, but the final decision to admit a patient was the responsibility of the physician in charge of the hospital ward.

#### Scores

qSOFA (one point each for systolic hypotension [≤100 mmHg], tachypnea [≥22/min] or altered mentation [Glasgow coma score ≤14]) and SOFA scores as well as SIRS criteria were assessed at inclusion. We used a modified version of the SOFA score developed for resource-limited settings, as previously described [16]. SpO_2_ was used instead of PaO_2_/FIO_2_ and the systolic arterial blood pressure instead of the mean arterial blood pressure. For calculation of the SOFA score, missing values were assumed to be within the normal range (i.e. assigning a 0 value). Considering that bilirubin was not tested and considered normal in outpatients, a variable of the SOFA score was missing in 22 patients (9 platelets count, 11 creatinine, 5 bilirubin). Dichotomic variables (<2 and ≥2) were generated for every score.

### Main outcome and statistical analysis

The outcome was 28-day all-cause mortality. Demographic, clinical, laboratory and scores characteristics as well as infectious diagnoses of survivors and non-survivors were compared using Wilcoxon-Mann-Whitney and chi-square tests. The prognostic accuracy of the different scores to predict 28-day mortality was assessed by comparing the area under the receiver operating characteristic curve (AUROC) calculated for each score. An AUROC between 0.6 and 0.7 was considered as poor, adequate between 0.7 and 0.8, good between 0.8 and 0.9 and excellent above 0.9 [4]. We also compared the survival rate of patients with less than 2 versus 2 points or more for SOFA and qSOFA scores or with less than 2 versus 2 criteria or more for SIRS. The diagnostic performances (sensitivity, specificity, negative and positive predictive values) of qSOFA, SOFA and SIRS were calculated using the same cutoffs. The association between the categorical variables of qSOFA, SOFA and SIRS and 28-day all-cause mortality was evaluated by stratified analyses. The sample size of the study, which was primarily conducted to identify the causes of fever, guarantees a power of 70% to detect an absolute increase in mortality of 10% in patients with a qSOFA score of 2 or more compared to the overall baseline mortality rate which was fixed at 6% according to our data [6]. All analyses were performed with STATA software (version 12, Stata Corp, College Station, TX, USA). Differences were considered as statistically significant with a two-sided P value of less than 0.05.

### Ethical considerations

All participants consented in writing to participation in this research. The Ifakara Health Institute Review Board (IHI/IRB/No: 12–2013), the Medical Research Coordinating Committee of the National Institute for Medical Research (NIMR/HQ/R.8a/Vol. IX/1561) of Tanzania and the Ethics Committee of the canton of Basel of Switzerland (Ref. Nr. EK: 1612/13) gave ethical clearance.

## Results

**Fig 1:**
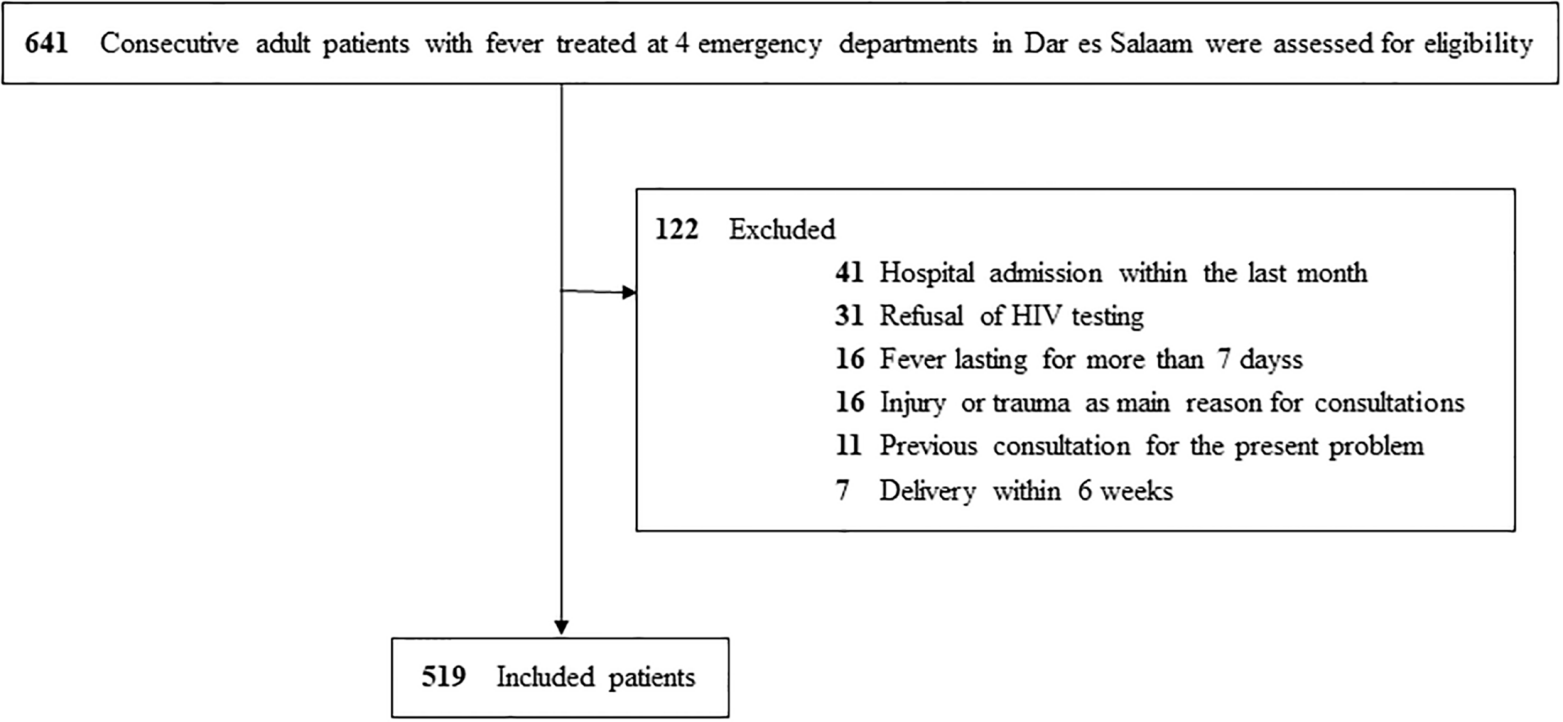
Flowchart of eligible patients and exclusion criteria.

A total of 641 patients with fever were prospectively enrolled in the study and assessed for eligibility. After exclusion of 41 patients due to hospitalization in the last month, 31 to HIV screening refusal, 16 to fever for more than 7 days, 16 because their main complaint was an injury or trauma, 11 because it was not their first consultation for the present symptoms and 7 because they were within 6 weeks of delivery, 519 patients were included in the analyses (Fig 1). Among 16022 patients attending the outpatient clinic of the hospitals, including consultations for chronic diseases, who were screened for the presence of fever between the 23^rd^ of September 2013 (data not available during the first two months of the study) and the end of recruitment on May 14^th^ 2014, 556 (3.5%) had documented fever.

Characteristics of the study population are shown in Table 1. The median age was 30 years, 47% were females and the HIV prevalence was 25%. Among the 519 included patients, 441 (85%) had a microbiologically (positive result of any kind of laboratory test aiming at detecting a pathogen (culture, molecular, serology, rapid diagnostic test)) or clinically documented infection and 78 (15%) an unknown cause of fever. Among these 441 patients with documented infection, 628 diagnoses were clinically and/or microbiologically identified. Among patients with a clinically-documented infection, the sites of infection were the respiratory tract (N = 223; 43%), the genito-urinary tract (N = 38; 7%), the abdomen (N = 31; 6%), the central nervous system (N = 5; 1%), the skin (N = 2; 0.4%) and the skeletal system (N = 1; 0.2%). Among patients with a microbiologically-documented infection, most common diagnoses were dengue (N = 134; 26%), malaria (N = 29; 6%) and typhoid fever (N = 28; 5%).

**Table 1:** Baseline characteristics of study participants according to survival at day 28.

	All N = 519	Day 28 non-survivors N = 32	Day 28 survivors N = 487	
	No. (%) or Median (IQR)			p-value
Age, years	30 (23–40)	41 (28–49)	29 (23–39)	<0.001
Female sex	246 (47%)	19 (59%)	227 (47%)	0.16
Inclusion in a hospital (versus healthcare center)	343 (66%)	30 (94%)	313 (64%)	0.001
HIV positive	128 (25%)	18 (56%)	110 (23%)	<0.001
CD4, cells/mm^3^ (HIV+ patients)	122 (51–260)	80 (45–148)	127 (59–275)	0.07
Temperature, °C	38.3 (38.0–39.0)	38.4 (38.1–38.9)	38.3 (38.0–39.0)	0.63
Systolic blood pressure, mmHg	118 (106–126)	106 (98–120)	119 (106–126)	0.001
Systolic blood pressure ≤100 mmHg	89 (17%)	14 (44%)	75 (15%)	<0.001
Respiratory rate, breaths/min	23 (20–26)	28 (26–32)	23 (20–25)	<0.001
Respiratory rate ≥22/min	325 (63%)	27 (84%)	298 (61%)	0.01
Heart rate, beats/min	106 (90–119)	126 (111–133)	105 (90–117)	<0.001
Glasgow Coma scale score ≤14	17 (3%)	14 (44%)	3 (0.6%)	<0.001
**Laboratory results**				
White blood cell count, cells/mL	5.8 (4.2–8.6)	6.4 (5.2–13)	5.8 (4.2–8.5)	0.06
Platelets, 10^3^/μL ^a^	184 (118–252)	181 (85–292)	184 (122–252)	0.66
Creatinine, mg/dL ^b^	0.99 (0.84–1.2)	1.0 (0.80–1.6)	0.99 (0.84–1.2)	0.26
Bilirubin, mg/dL ^c^	0.32 (0.16–0.72)	0.15 (0.08–0.23)	0.34 (0.18–0.81)	<0.001
**Sites of infection**				
Respiratory	223 (43%)	18 (56%)	205 (42%)	0.12
Genito-urinary	38 (7%)	3 (9%)	35 (7%)	0.65
Abdominal	31 (6%)	4 (13%)	27 (6%)	0.11
Other sites	8 (2%)	5 (16%)	3 (0.6%)	<0.001
**Most common microbiologically documented infections**				
Dengue	134 (26%)	2 (6%)	132 (27%)	0.01
Malaria	29 (6%)	0 (0)	29 (6%)	0.16
Typhoid	28 (5%)	0 (0)	28 (6%)	0.16
**SIRS**				*0*.*21*
0	30 (6%)	0 (0)	30 (6%)	
1	65 (13%)	3 (9%)	62 (13%)	
2	126 (24%)	6 (19%)	120 (25%)	
3	215 (41%)	14 (44%)	201 (41%)	
4	83 (16%)	9 (28%)	74 (15%)	
SIRS ≥2	424 (82%)	29 (91%)	395 (81%)	0.18
SOFA ≥2 ^d^	156 (30%)	23 (72%)	133 (27%)	<0.001
**qSOFA** ^e^				*<0*.*001*
0	172 (33%)	1 (3%)	171 (35%)	
1	268 (52%)	12 (38%)	256 (53%)	
2	74 (14%)	14 (44%)	60 (12%)	
3	5 (1%)	5 (16%)	0 (0)	
qSOFA ≥2	79 (15%)	19 (59%)	60 (12%)	<0.001
**Management**				
Antibiotic therapy	210 (41%)	24 (75%)	186 (38%)	<0.001
Hospital admission	81 (16%)	31 (97%)	50 (10%)	<0.001
Length of hospital stay ^f^ (admitted patients)	4 (2–8)	3 (2–12)	4 (2–7)	0.66

^a^ 9 missing values;

^b^ 11 missing values;

^c^ 329 missing values;

^d^ Considering that bilirubin was not tested and considered normal in outpatients, a variable of the SOFA score was missing in 22 patients (9 platelets count, 11 creatinine, 5 bilirubin);

^e^ Variables for the calculation of qSOFA were available for every patient;

^f^ 1 Length of hospital stay missing.

Overall, 210 (40%) patients received antibiotics following a pre-defined management algorithm and 16% were admitted to the hospital. Fifteen percent of patients had a qSOFA of 2 or higher, 30% a SOFA of 2 or higher and 82% a SIRS of 2 or higher. Nine (1.7%) patients died within 2 days, 18 (3.5%) within 7 days and 32 (6.2%) within 28 days. Among the 32 non-survivors, 42 infectious diagnoses were established (12 acute respiratory infections, 6 fevers of unknown origin, 5 cryptococcal infections, 4 tuberculosis, 4 gastroenteritis, 3 urinary tract infections, 2 dengue, 1 meningitis, 1 measles, 1 malaria, 1 primary bacteremia (*Streptococcus pyogenes*), 1 non-streptococcal pharyngitis, 1 diabetic foot infection). Among patients who died within 28 days, one was not admitted, 25 died in the hospital after a mean of 6 days (SD 6) and six died after a mean of 8 days (SD 7) after hospital discharge. Patients who died were older (median age of 41 versus 29 years; p<0.001), were more often HIV-infected (56% versus 23%; p<0.001), were mainly recruited in hospitals and not in healthcare centers (94% versus 64%; p = 0.001) and were less often infected by dengue virus (6 versus 27%; p = 0.01) (Table 1).

Hospital admission rates and 28-day all-cause mortality according to qSOFA, SOFA and SIRS are reported in Table 2. Mortality was 3% for patients with a qSOFA score of less than 2 and 24% for those with a score of 2 or more (absolute difference, 21%; 95% CI 12–31%; p<0.001). It was 3% for patients with an increase of SOFA score of less than 2 points and 15% for those with an increase of more than 2 points (absolute difference, 12%; 95% CI 7–18%; p<0.001). In contrast, the mortality rate was not significantly different between patients who had less than 2 SIRS criteria and those who had 2 and more (absolute difference, 4%; 95% CI -0.6—+8%, p = 0.18).

**Table 2:** Hospital admission rate and mortality at day 28 according to scores.

	qSOFA score			SOFA score			SIRS score		
	<2 points N = 440	≥2 points N = 79	Absolute difference	<2 points N = 363	≥2 points N = 156	Absolute difference	<2 criteria N = 95	≥2 criteria N = 424	Absolute difference
	No. (%)	No. (%)	% (95% CI)	No. (%)	No. (%)	% (95% CI)	No. (%)	No. (%)	% (95% CI)
Hospital admission	40 (9)	41 (52)	43 (32–54)	33 (9)	48 (31)	22 (14–30)	6 (6)	75 (18)	11 (5–18)
Day 28 mortality	13 (3)	19 (24)	21 (12–31)	9 (3)	23 (15)	12 (7–18)	3 (3)	29 (7)	4 (-0.6–8)

Compared to patients with a qSOFA score below 2, those with a score of 2 or greater had a 10-fold odds increased risk in 28-day mortality. Compared to patients with a SOFA score and a SIRS below 2, those with a score of 2 or greater had 6-fold and a 2-fold odds increase in 28-day mortality, respectively (Table 3). The OR for qSOFA was much lower in HIV positive (5; 95% CI 2–13) than in HIV negative patients (17; 5–54), which was not the case for SOFA and SIRS. Stratified analyses by the other factors did not show significant differences.

**Table 3:** Diagnostic performances of the different scores using a cut-off or 2 or greater to predict day 28 mortality.

	qSOFA	SOFA	SIRS
Sensitivity, % (95% CI)	59 (41–76)	72 (53–86)	91 (75–98)
Specificity, % (95% CI)	88 (84–91)	73 (69–77)	19 (16–23)
Predictive value, % (95% CI)			
Positive	24 (15–35)	15 (9.6–21)	7 (4.6–9.7)
Negative	97 (95–98)	98 (95–99)	97 (91–99)
Likelihood ratio (95% CI)			
Positive	4.8 (3.3–7.0)	2.6 (2.0–3.4)	1.1 (1.0–1.3)
Negative	0.5 (0.3–0.7)	0.4 (0.2–0.7)	0.5 (0.2–1.5)
Odds ratio (95% CI)	10 (5–22)	7 (3–15)	2 (0.7–8)

A qSOFA and SOFA scores of 2 or greater had a sensitivity of 59% and 72% and a specificity of 88% and 73% for predicting 28-day mortality. The sensitivity of SIRS was higher (91%), but the specificity was poor (19%) (Table 3). The negative predictive value for 28-day mortality of a qSOFA, SOFA and SIRS below 2 was very high for all scores (97, 98 and 97%, respectively).

**Fig 2:**
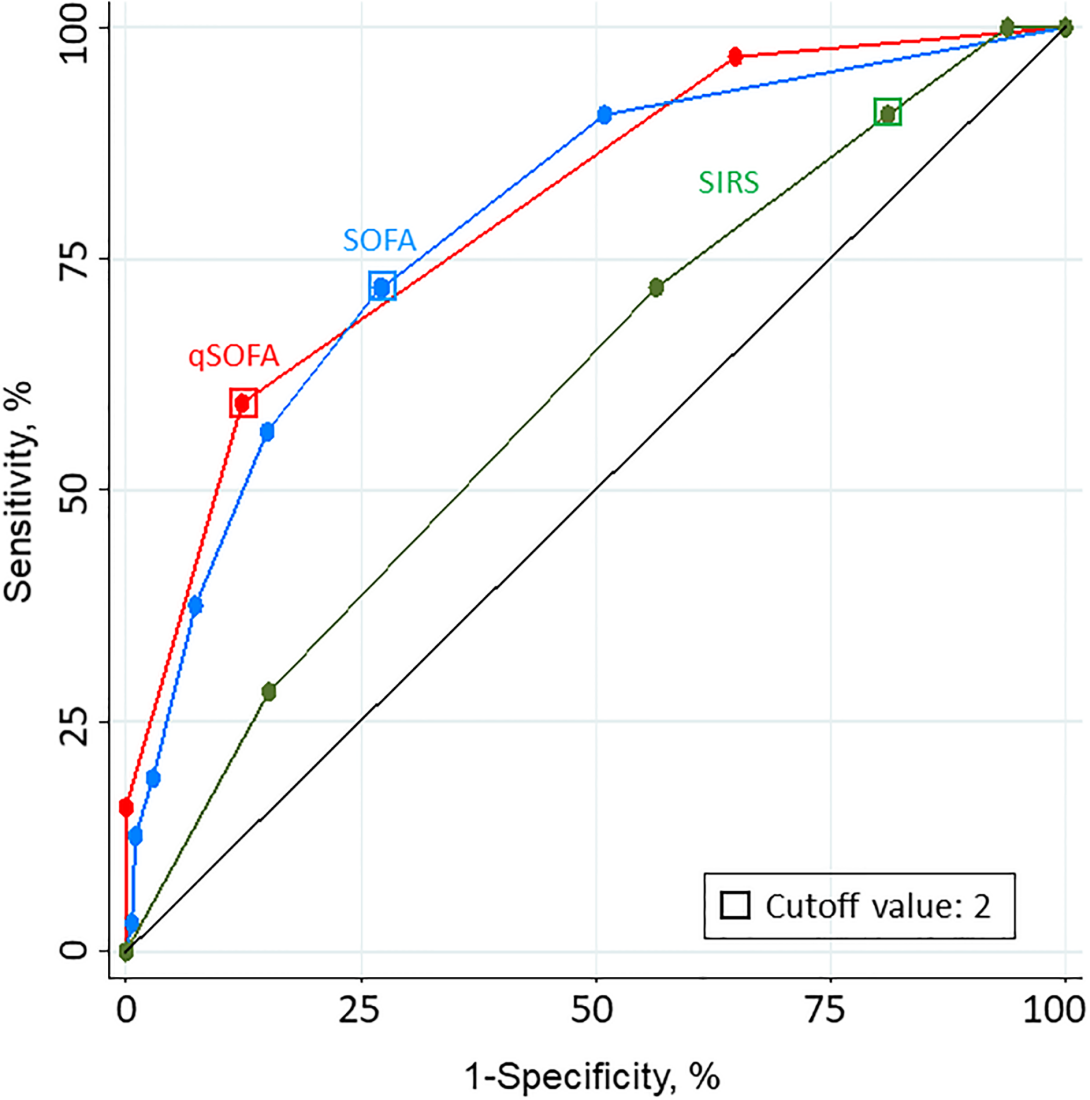
Receiver operating characteristic (ROC) curves for 28-day mortality. SOFA indicates Sequential (Sepsis-related) Organ Failure Assessment; qSOFA, quick SOFA; SIRS, systemic inflammatory response syndrome. The area under the ROC curves (AUROC) for qSOFA is 0.80 (95% CI 0.73–0.87), SOFA 0.79 (95% CI 0.71–0.87) and SIRS 0.61 (95% CI 0.52–0.71).

As shown in Fig 2, the prognostic accuracy for 28-day mortality using qSOFA (AUROC = 0.80; 95% CI 0.73–0.87) and SOFA (0.79; 0.71–0.87) was better than using SIRS (0.61; 0.52–0.71) (p<0.01 for both pairwise comparisons with SIRS). qSOFA predicted 28-day mortality better in HIV negative (0.85; 0.76–0.93) than in HIV positive patients (0.72; 0.59–0.84), while the predictive accuracy of SOFA for 28-day mortality was not affected by HIV status (0.78; 0.67–0.90 in HIV negative and 0.72; 0.59–0.84 in HIV positive patients).

SIRS predicted 28-day mortality adequately in HIV negative patients (0.72; 0.57–0.87), but was not predictive of mortality in HIV positive patients (0.48; 0.36–0.61) (S1 Fig).

## Discussion

In a large cohort of febrile patients attending emergency departments of a resource-limited African country with high HIV prevalence, we investigated the prognostic accuracy of qSOFA, SOFA and SIRS as predictors of 28-day all-cause mortality. Among 519 patients of whom 85% had a microbiologically or clinically documented infection, qSOFA had a prognostic accuracy for 28-day mortality similar to that of SOFA but greatly superior to that of SIRS.

The robust prognostic accuracy of qSOFA (AUROC of 0.80) confirms and extends to emergency departments of an African urban environment previous analogous findings with respect to in-hospital mortality obtained in large North-American and European data sets (AUROC of 0.81 and 0.80) [4, 7]. Of note, a retrospective study including 329 patients with fever or hypothermia and SIRS admitted to a hospital (rather than attending the ED as in our study) in Gabon also showed a good predictive validity of qSOFA for in-hospital mortality with an AUROC of 0.83 [17]. The characteristics of the patients included in Gabon were similar to those included in our study with regard to age, sex, HIV prevalence and mortality. In the study conducted in Gabon, the sample size was limited and the prognostic accuracy of qSOFA could not be compared to that of SOFA as it was not calculated, nor to that of SIRS as only patients with 2 or more SIRS criteria were included.

In the present study, 82% of patients had a SIRS of 2 or greater while only 15% had a qSOFA of 2 or greater. SIRS criteria had a higher sensitivity (91%) compared to qSOFA score (59%). However, the discriminative value of SIRS score was poor with a very low specificity compared to qSOFA (19% versus 88%). Importantly, there was no difference in the rate of false-negatives between qSOFA and SIRS (3.0% versus 3.2%) with a high negative predictive value for both scores. These results are similar to those found in the study conducted in European emergency departments discussed above which support the use of qSOFA as a triage tool in emergency departments [7].

Interestingly, the mortality of patients with a SOFA score of 2 or more was 15%, which also is very well in line with the anticipated overall mortality of around 10% in this subset of patients according to the new Sepsis-3 definition [6]. In the present study, the mortality rate of patients with a qSOFA of 2 or greater was 24%, which is the same than the observed mortality in the prospective European emergency department study [7]. This observation supports the use of qSOFA as a bedside tool to identify among febrile patients the subgroup of septic patients with poor outcome. The performance characteristics of qSOFA (intermediate sensitivity and high specificity) make it an attractive triage instrument to assist clinicians in making the best use of limited resources.

The performance of qSOFA to predict mortality was much lower in HIV positive patients while the performance of SOFA was not affected by HIV status. The rate of false negative qSOFA scores was higher in HIV positive patients compared to HIV negative patients (1.4% versus 8.5%, p = 0.002). Additional studies including a higher number of HIV infected patients would help to better understand the performance of the different scores in this specific population.

This study has several strengths. First, it validates the discriminative power of qSOFA as a triage tool at emergency departments of a large African city in patients with sepsis due or related to infections that are quite different (malaria, dengue, typhoid; high HIV prevalence) from that found in Western countries. Second, it supports the use of qSOFA as a simple and accurate triage tool in a resource-limited setting in Africa. Indeed, we used the qSOFA calculated at admission and not the worse qSOFA value during the stay in the emergency department. Third, we followed up the patients to have 28-day mortality and not only in-hospital mortality [7]. This is especially relevant in a resource-limited setting where patients are likely to be discharged early for socio-cultural or economic reasons (median length of hospital stay of 4 days compared to 7 days in the prospective European emergency department study). Fourth, we made a lot of efforts to have a precise and broad clinical and microbiological documentation of infection. One limitation of the present study is that we did not measure lactate. However, when added to qSOFA in the analysis of large databases of admitted patients as well as in a cohort study of patients in emergency departments with a suspected infection, it did not perform better than without lactate [4, 7]. A second limitation is the few data that were missing to calculate the SOFA score, which may have affected its performance.

## Conclusions

Among patients with fever attending emergency departments in Dar es Salaam (urban Tanzania), the performance of qSOFA to predict 28-day mortality was not significantly different from a more sophisticated, but difficult to obtain SOFA score and it was greatly superior to SIRS. The qSOFA tool can be easily implemented in low-resource countries as it relies on simple clinical measurements and can be rapidly calculated at the patient side without the need of blood tests. These findings support the use of qSOFA as a triage tool in emergency departments of resource-poor countries to identify patients with infection at higher risk of mortality who need prompt management and a higher surveillance level. This is particularly critical in health care settings where patient triage is often done by nurses given the limited number of medical doctors.

## Supporting information

S1 Fig Receiver operating characteristic (ROC) curves for 28-day mortality in (A) HIV negative and (B) HIV positive patients. SOFA indicates Sequential (Sepsis-related) Organ Failure Assessment; qSOFA, quick SOFA; SIRS, systemic inflammatory response syndrome. In HIV negative patients, the area under the ROC curves (AUROC) for qSOFA is 0.85 (95% CI 0.76–0.93), SOFA 0.78 (95% CI 0.67–0.90) and SIRS 0.72 (95% CI 0.57–0.87). In HIV positive patients, the area under the ROC curves (AUROC) for qSOFA is 0.72 (95% CI 0.59–0.84), SOFA 0.72 (95% CI 0.59–0.84) and SIRS 0.48 (95% CI 0.36–0.61).
(TIF)

S1 Table Diagnostic tests performed in the study population. (DOCX)
