# Supplementary figures and images for: Prognostic value of quickSOFA as a predictor of 28-day mortality among febrile adult patients presenting to emergency departments in Dar es Salaam, Tanzania
Source: PLoS One. 2018 Jun 14;13(6):e0197982. doi: 10.1371/journal.pone.0197982 (PMC6002058; doi:10.1371/journal.pone.0197982)

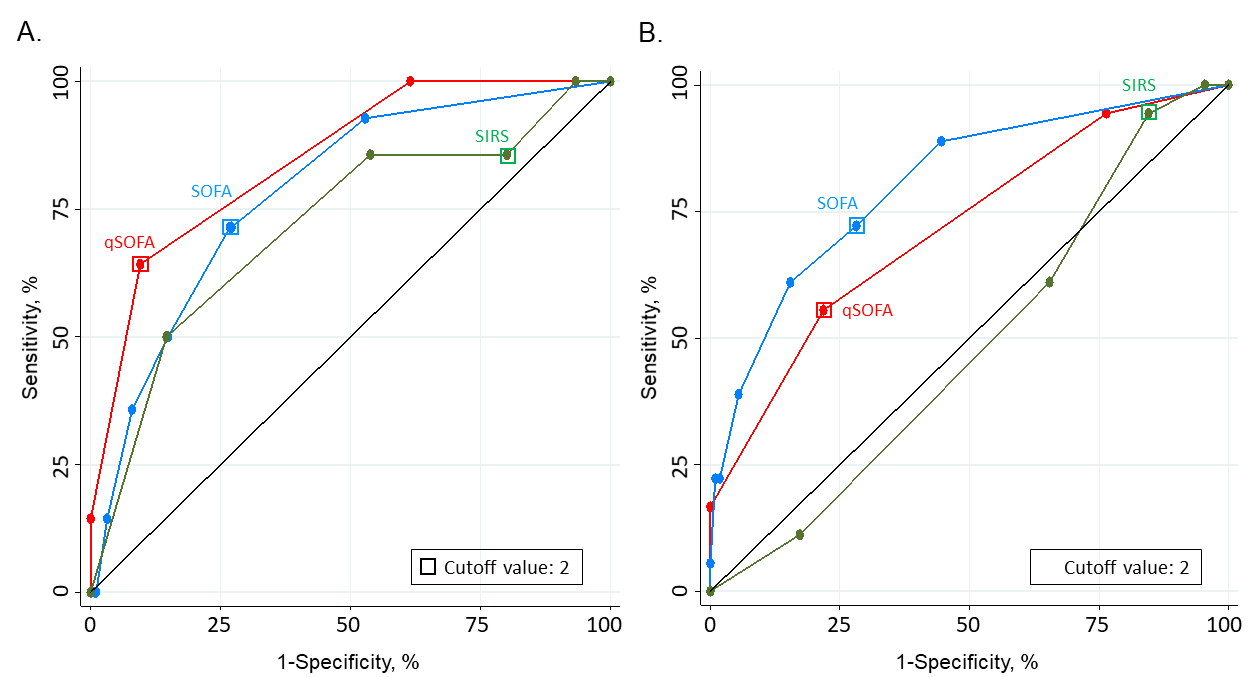

Supplement: S1 Fig — SOFA indicates Sequential (Sepsis-related) Organ Failure Assessment; qSOFA, quick SOFA; SIRS, systemic inflammatory response syndrome. In HIV negative patients, the area under the ROC curves (AUROC) for qSOFA is 0.85 (95% CI 0.76–0.93), SOFA 0.78 (95% CI 0.67–0.90) and SIRS 0.72 (95% CI 0.57–0.87). In HIV positive patients, the area under the ROC curves (AUROC) for qSOFA is 0.72 (95% CI 0.59–0.84), SOFA 0.72 (95% CI 0.59–0.84) and SIRS 0.48 (95% CI 0.36–0.61). (TIF) [file pone.0197982.s001.tif]
