# Supplementary material for: Prognostic value of quickSOFA as a predictor of 28-day mortality among febrile adult patients presenting to emergency departments in Dar es Salaam, Tanzania
Source: PLoS One. 2018 Jun 14;13(6):e0197982. doi: 10.1371/journal.pone.0197982 (PMC6002058; doi:10.1371/journal.pone.0197982)
Supplement: S1 Table — (DOCX) [file pone.0197982.s002.docx]

**S1 Table. Diagnostic tests performed in the study population.**

| **Tests performed in all patients** | | |
| --- | --- | --- |
|  | | HIV rapid diagnostic test |
|  | | Malaria rapid diagnostic test and if positive, blood slide |
|  | | Typhoid rapid diagnostic test |
|  | | Blood cultures |
|  | | Real-time multiplex PCR for tropical pathogens in blood |
|  | | Real-time multiplex PCR for respiratory pathogens in naso-pharyngeal sample |
|  | |  |
| Additional investigations in patients with **cough or dyspnea, and at least 1 symptom/sign among tachypnea, pleuritic chest pain or abnormal chest auscultation** | | |
|  | | Chest X-ray |
|  | |  |
| Additional investigations in patients with **cough lasting ≥ 2 weeks or in patients with cough and a chest X-ray suggestive of tuberculosis or in HIV infected patients with cough of any duration** | | |
|  | | TB screening: 2 sputa for GeneXpert MTB/RIF |
|  | |  |
| Additional investigations in patients with **cough lasting ≥ 2 weeks or in HIV infected patients with cough of any duration** | | |
|  | | Histoplasma antigen in urine and Histoplasma IgM in serum |
|  | |  |
| Additional investigations in patients **with a negative HIV rapid test** | | |
|  | Serum p24 antigen | |
|  | | |
| Additional investigations in patients **infected with HIV** **and CD4 count <100 cells/mm^3^** | | |
|  | | Serum Cryptococcus antigen |
|  | |  |
| Additional investigations in patients with **cough or dyspnea infected with HIV and CD4 count <200 cells/mm^3^** | | |
|  | | Immunofluorescence and *Pneumocystis jiroveci* PCR on induced sputum |
|  | | Serum β-D-glucan |
|  | |  |
| Additional investigations in patients with **diarrhea** (≥ 3 loose or liquid stools) | | |
|  | | Stool examination for *Entamoeba* spp |
|  | | Stool culture for *Salmonella*, *Campylobacter* and *Shigella* |
|  | | Real-time multiplex PCR for gastro-intestinal pathogens in stool |
|  | | Rapid test for rotavirus and norovirus in stool |
|  | |  |
| Additional investigations in patients with **tonsillitis** | | |
|  | | *Streptococcus pyogenes* rapid test |
|  | |  |
| Additional investigations in patients with **neck stiffness** | | |
|  | | Cerebrospinal fluid analyse and culture |
|  | |  |
| Additional investigations in patients with **dysuria and/or polyuria** | | |
|  | | Urine dipstick and, if positive for leucocytes or nitrites, urine culture |
|  | |  |
| Additional investigations in patients with **urethral discharge (men) and/or dysuria** | | |
|  | Urine dipstick and, if positive for leucocytes, PCR for *Chlamydia trachomatis* and *Neisseria gonorrhoea* in urine | |
|  | | |
| Additional investigations in patients with **vesicles and/or ulcers in the genito-anal area** | | |
|  | Herpes simplex virus PCR in a swab and rapid test for syphilis | |
|  | | |
| Addtional investigations in patients with **anorectal pain with tenesmus and discharge** | | |
|  | *C. trachomatis* and *N. Gonorrhoea* PCR on rectal swab | |
|  | | |
| Additional investigations in patients with **scrotal swelling or tenderness without rotation of the testis and history of trauma** | | |
|  | Urine dipstick and, if positive for leucocytes, PCR for *C. trachomatis* and *N. gonorrhoea* in urine | |
|  | | |
| Additional investigations in patients with **genital ulcer and/or maculo-papular rash involving palms and/or soles** | | |
|  | Rapid test for syphilis | |
|  | | |
| Additional investigations in patients with a **dermatomal vesicular rash** | | |
|  | Herpes zoster virus PCR in a vesicule swab | |
|  | | |
| Additional investigations in patients **with elevated liver enzymes (3-fold increase in ALT) and without definitive diagnosis at inclusion** | | |
|  | Serologies for hepatitis A, B, C | |
|  | | |
| Additional investigations in patients **without definitive diagnosis at inclusion** | | |
|  | | Serologies for Ebstein Barr virus, Cytomegalovirus, *Toxoplasma gondii*, *Histoplasma capsulatum*  PCR for Rift Valley fever |
